# Equestrian Injury Presentations to a Regional Trauma Centre in Ireland

**DOI:** 10.1155/2018/7394390

**Published:** 2018-06-03

**Authors:** A. Abdulkarim, A. Juhdi, P. Coffey, Lily Edelson

**Affiliations:** ^1^Department of Trauma and Orthopaedic Surgery, Midland Regional Hospital, Tullamore, Ireland; ^2^Royal College of Surgeons, Dublin, Ireland; ^3^Department of Orthopedic Surgery, Our Lady's Hospital, Navan, Ireland; ^4^University of limerick, Limerick, Ireland

## Abstract

**Background:**

The Irish Equestrian industry provides over 12,500 full time job equivalents contributing in excess of €454 million to the Irish economy annually. For such an important industry there is a paucity of information relating to equestrian injuries.

**Aims:**

The aim of this study was to determine the demographics, characteristics, and outcomes of equestrian related injuries presenting to the emergency department of a regional trauma centre in Ireland over the course of one year.

**Methods:**

Retrospective analysis of all 30,700 presentations to the emergency department (ED) of the Midland Regional Hospital (MRH) Tullamore in 2013 was performed to identify equestrian related presentations. Patient demographics, mechanism of injury, radiology results, management, and follow-up data were collected and analysed using Microsoft Excel software.

**Results:**

A total of 149 equestrian related presentations were identified during the study period. There were significantly more females involved in equestrian injuries than males (58% versus 42%). Falling from a horse contributed to significantly more presentations and admissions than any other cause. 36% of presentations were associated with a radiological abnormality. Types of injuries identified included skeletal fractures (27.5%), joint dislocation/subluxation (5%), concussion (12.5%), and splenic laceration/intraperitoneal haemorrhage (1%). Admission or transfer to tertiary care was required for 18% of equestrian injuries. Only 43% of presentations were discharged back to primary care from the emergency department.

**Conclusion:**

This study identifies a high incidence of morbidities associated with equestrian presentations. In addition we recognised populations at risk of specific injuries and described high-risk mechanisms of injury.

## 1. Introduction

The equestrian industry is an important source of employment in Ireland. It contributes an estimated €454 million to the Irish economy [1]. The industry is believed to provide 12,512 full time job equivalents [1]. Economically, the breeding sector is the largest, followed by the competition and recreational sectors, respectively [1]. The Midland region of Ireland's share of the national sports horse herd is increasing, with an estimated 8,500 horses [2]. The Midland Regional Hospital (MRH) Tullamore caters for the Kilbeggan racecourse as well as many equestrian businesses offering recreational services.

The role of the horse in Irish society has changed dramatically in the last century, with its primary use now being sports and recreational activities. It is estimated that 1.33% of the Irish population are regularly involved with sports horses [2]. The combination of a horse's strength, height, and unpredictability can lead to significant injuries to those who engage in equestrian related activities. Equestrian activities were responsible for over 40% of sports related spinal injuries admitted to the Irish National Spinal Injuries Unit over a ten-year period [3]. One study looking at professional jump racing did find a lower rate of injury in the Irish professional race industry compared to that in the UK [4]. A review of the literature revealed that there is a paucity of research investigating equestrian related emergency department presentations in Ireland, particularly in relation to recreational riders.

This study was designed to assess equestrian related injury presentations to emergency departments. It is a retrospective study examining the mechanisms, nature, and outcomes of equestrian injuries presenting to the MRH Tullamore, Co. Offaly. There were a total of 30,700 attendances to the MRH emergency department for 2013.

## 2. Methods

The MRH Tullamore is the trauma referral centre for the midlands region of Ireland with a population of 282,410 [5]. Every presentation to the Emergency Department at MRH from the 1st of January 2013 to the 31st of December 2013 was assessed retrospectively to determine if an injury had been sustained in an environment related to the equestrian industry. Triage notes of all 30,700 presentations to the department were reviewed to determine if a presentation may be equestrian related and further chart analysis was used for confirmation. Patient demographics, date of occurrence, mechanism of injury, radiology reports, management, and follow-up data were collected and analysed. Radiology reports were available for all imaging and the imaging was reported by consultant radiologists. Statistical analyses were performed using Microsoft Excel.

## 3. Results

The Emergency Department at MRH Tullamore received a total of 30,700 presentations between 1 January and 31 December 2013. One hundred and forty-nine equestrian related presentations were identified. From these presentations, 189 injuries were identified. No mortalities were identified. Females accounted for more presentations than males (58% versus 42%) (Table 1). The median and mean age were 25 and 27, respectively (range 5 to 77). Eighty-two per cent (82%) of those injured were recreational horse riders.

The most common mechanism of injury was a fall from horseback, contributing to almost 80% of all presentations (Table 2). Other important mechanisms included kicks, trampling, and bites. Soft tissue injuries were the most common type of injury (42%), followed by fractures (27.5%), head injuries (12.5%), and joint dislocation/subluxation (5%) (Tables 1 and 2). The upper limb was the most common site of injury identified. The distribution and associated mechanism of injuries are shown in (Table 2). Of the total equestrian related presentations, 36% had radiological abnormalities. The radiological abnormalities included upper limb fractures (32%), clavicular fractures (19%), lower limb fractures (15.5%), spinal fractures (12.5%), dislocations (11%), and pelvic fractures (4%).

Both Tables 3 and 4 demonstrate the difference in injuries distribution and outcomes between recreational and professional riders.

Following presentation to the emergency department, 43% were discharged back to primary care, 39% were referred to the outpatients department, 16% required admission, and 2% were transferred to tertiary care (Table 1). 80% of admissions were secondary to falls from a horse. Head injuries accounted for 12.5% of admission. All had a Glasgow Coma Scale of 15 on arrival in the emergency department. One-third of patients with head injuries had imaging; the remainder were discharged after 24 hours of neurological observations. One individual had a fracture of his maxilla and floor of orbit. No intracranial pathology was identified.

Seventy-seven per cent (77%) of admissions had a fracture. Five patients required surgical intervention. One fractured femur and two ankle fractures required open reduction and internal fixation. Percutaneous fixation was required for a fractured ulna and radius. A wound washout was required for an open digital fracture. Six patients were admitted with spinal fractures, 3 had stable fractures at the thoracolumbar junction, and 2 had stable upper thoracic spine fractures. One patient had an unstable lower cervical spine fracture with neurological deficits, requiring transfer to the Irish National Spinal Injuries Unit for acute spinal stabilisation.

There were three injuries requiring admission that were not related to a fall from a horse. One individual was admitted after a bite injury resulting in a nipple amputation. Two admissions were secondary to kicks from a horse, one requiring neurological observations for a head injury and the other requiring ICU admission under the general surgical team for conservative management of a splenic laceration.

## 4. Discussion

This study has revealed equestrian related presentations carry a high risk of injury, with associated morbidity, requiring the utilization of significant hospital resources. Equestrian injuries accounted for 0.49% of presentations to the emergency department in the study period. Previous studies have shown that horse riding is as hazardous, if not more hazardous, as other sports including soccer, rugby, skiing, and motorcycle riding per hour of participation [6, 7]. Figures from the US consumer products safety commission for the year 2014 showed that injuries from horse riding were only 28,670 injuries which is much less when compared to other sport like cycling with 346,208 injuries, American football with 237,392 injuries, soccer with 141,929 injuries, and boxing and martial arts with 52,217 injuries [8]. On the other hand equestrian riding is considered to have amongst the highest mortality rates of all sports with an annual death rate of 1 per 1 million populations [9].

In agreement with other authors our results showed that those involved in equestrian related injuries in this study were most commonly female, recreational horse riders, akin to their international counterparts [3, 10–13]. Recreational riders were more likely to have sustained a fracture than professional jockeys, in addition to this they were more likely to require surgical intervention; however admission rates were comparable between recreational riders and professional jockeys. Given the pedigree of horses involved in professional sport, it could be expected that professional jockeys would be more likely to be involved in accidents which involve greater velocities and forces than those involving recreational riders. This may indicate that their experience and training enables them to limit injury when accidents occur, a concept that is supported by the literature [13–15].

Injury distribution in this study was similar to that reported in studies from other jurisdictions [11, 12, 15–17]. Soft tissue injuries in isolation were the predominant injury type identified with upper limb and pectoral girdle the anatomical regions in which those presenting to the emergency department were most likely to be injured. Fractures comprised only 27.5% of injuries; however 77% of those that required admission to hospital had suffered a fracture. Head injuries made up 12.5% of injuries (and were present in 22% of admission) which was double the rate reported in the only comparable study identified in the literature [18]. The study by Khan et al. [18], looking at demographics of horse riding injuries in southeastern Ireland, was undertaken 10 years prior to this study and the difference in head injury rates may reflect either changes in practice amongst riders or more likely a greater awareness and diagnosis of mild traumatic brain injuries such as concussion.

Consistent with the literature, falls are the prevailing mechanism of injury in equestrian presentations [10–12, 16]. While falls are inevitable to some degree in equestrian activities, they account for the highest morbidity in the sport. We found that falls from a horse also were responsible for the most significant injuries, highest number of admissions, and surgical interventions.

Suggestions to potentially reduce the incidence and morbidity of equestrian related injury can be focused specifically on the rider, on protective equipment, and on prevention of secondary injury. In the case of riders, research has shown that less experienced riders are at greater risk of injury [12, 14, 15] with a substantial decline in injury achieved after approximately 100 hours of experience [14]. Specific training should be provided on how to manage a fall, including releasing ones feet from the stirrups, positioning to limit injury when hitting the ground, and how to roll away to avoid being potentially kicked from the horse after a fall. Many of the kick injuries recorded in this study were after a fall from a horse. Protective equipment such as helmets and body protectors are mandatory in many competitive and professional equestrian activities in Ireland [19, 20] but their use is not as strictly enforced in recreational equestrian activities. Despite being shown to reduce the frequency and severity of head injuries, studies in other jurisdictions have shown a consistently low rate of helmet use with less than 40% of riders wearing a helmet at the time of injury [6]. A weakness of this study is that the use of protective equipment by those attending with equestrian injuries was not recorded, an area of particular paucity of data specific to Ireland. Equestrian events were responsible for over 40% of sports related attendances to the Irish National Spinal Unit [3] and given the relatively high frequency of spinal injuries basic medical training for riders and trainers should be encouraged in order to reduce the risk of secondary injury from inappropriate mobilisation after injury.

For clinicians, the potential forces involved and the broad nature of injuries identified in this study should give rise to a high index of suspicion for injury when dealing with equestrian related presentations to the emergency department. In our study 16% of presentations involved multiple injuries, which introduces the risk of more subtle injuries being overlooked because of a more obvious or distracting injury. As a result, it is imperative that patients suffering an equestrian related injury receive a thorough physical inspection, as per advanced trauma life support (ATLS) guidelines.

In Ireland, the contribution of the horse sport industry to the Irish economy is more than €816 million per annum. The competition sector accounted for €168 million expenditure in the sport horse sector while a total of €103 million was spent within the affiliated leisure sector [1]. Giving the above figures, injuries with horse riding represent a significant cost and that could be partly related to the cost of medical care associated with diagnosis and treatment of these injuries and partly due to time off participation from events and loss of income to the professional riders.

According to Turner et al., Jockeys are paid a fixed fee for every ride and a fixed percentage of any prize money achieved for winning the race or being placed (around 8% of the prize money). In 2005, the riding fee was £85.79 per ride on the flat and £117.15 for every jump ride. The ability and popularity of a jockey determine how many rides they are offered and the top jockeys may have up to 1000 rides in 12 months. The average jockey would expect to be booked for around 300 rides/year (flat) or 200 rides/year (jump), which is equivalent to an annual income in of £25,737 (flat) or £23,430 (jump). Turner et al. reported in their paper that the majority of horse related injuries they studied were minor and one-third of jockeys returned to racing within 2 weeks and 45% of the injuries resulted in payouts of £1000 [21].

Curry et al. studied The number and types of costs associated with compensation claims for nonfatal horse riding injuries and their figures demonstrated that the mean cost of a claim was 43,374 Australian dollars (AUD) in flat racing and AUD 52,589 in jumps racing. Also the claim incidence and median cost of a claim increased, with age. On average, jockeys were absent from work for 9 weeks following a substantive injury [22]. Although it is not possible to compare these figures to our results directly as no cost related data were collected, these figures are comparable to Ireland in general after adjustment for population and difference in the cost of health care between the two jurisdictions.

Strengths of this report are its large inclusion criteria, thus reducing selection biases. Patients were routinely followed up for all injuries and only discharged upon their resolution. Drawbacks of our study include its retrospective nature and the lack of data on the use of protective equipment.

## 5. Conclusion

The equestrian industry, despite its recreational benefit, employment, and economic contributions, is not without hazard. This study should help serve to develop safety awareness and promote public health strategies having identified specific populations at risk, as well as characterising injuries and mechanisms. Specific areas for future research have also been identified.

## Figures and Tables

**Table 1:** Demographics, type of injury, and outcome of equestrian related presentations.

	Number (%)
ED presentations	149
Sex	
Female	87 (58)
Male	62 (42)
Age	
0–18	51 (34)
18–65	97 (65)
>65	1 (1)
Outcome	
Discharge	64 (43)
Admission	24 (16)
Out patients dept.	58 (39)
Transfer	3 (2)
Injuries^a^	189
Injury type	
Soft tissue	80 (42)
Fracture	52 (27.5)
Dislocation	9 (5)
Head injury	24 (12.5)
Polytrauma	24 (12.5^b^)

^a^More than one injury per patient possible; ^b^% of ED presentations.

**Table 2:** Mechanism of injury and distribution.

	Upper limb	Lower limb	Pectoral girdle	Trunk	Spine	Head	Total mechanism (%)
Fall	44	15	19	19	23	12	132 (79)
Kick	9	2	5	8	0	11	35 (19)
Trample	0	9	0	0	1	0	10 (5)
Bite	2	0	0	1	0	1	4 (2)
Horseback	5	3	0	0	0	0	8 (4)

Total distribution (%)	60 (32)	29 (15.5)	24 (12.5)	28 (15)	24 (12.5)	24 (12.5)	

*N* = number of injuries out of 189 total injuries.

**Table 3:** Injury distribution between recreational and professional riders.

	Recreational riders	Professional rider
Fracture	44	8
Soft tissue injury	70	10
Dislocation	7	2
Head injury	18	6
Polytrauma	20	4

*Total*	*159*	*30*

**Table 4:** Comparison in outcome of presentation between recreational and professional riders.

	Recreational riders	Professional riders
Discharge	54	10
Admission	13	11
Out patients dept.	50	8
Transfer	3	0

*Total*	*120*	*29*
